# The effects of violet and blue light irradiation on ESKAPE pathogens and human cells in presence of cell culture media

**DOI:** 10.1038/s41598-021-04202-x

**Published:** 2021-12-28

**Authors:** Richard Bauer, Katharina Hoenes, Tobias Meurle, Martin Hessling, Barbara Spellerberg

**Affiliations:** 1 Institute of Medical Microbiology and Hygiene, University Hospital Ulm, 89081 Ulm, Germany; 2 Institute of Medical Engineering and Mechatronics, Ulm University of Applied Sciences, 89081 Ulm, Germany

**Keywords:** Antimicrobials, Cellular microbiology

## Abstract

Bacteria belonging to the group of ESKAPE pathogens are responsible for the majority of nosocomial infections. Due to the increase of antibiotic resistance, alternative treatment strategies are of high clinical relevance. In this context visible light as disinfection technique represents an interesting option as microbial pathogens can be inactivated without adjuvants. However cytotoxic effects of visible light on host cells have also been reported. We compared the cytotoxicity of violet and blue light irradiation on monocytic THP-1 and alveolar epithelium A549 cells with the inactivation effect on ESKAPE pathogens. THP-1 cells displayed a higher susceptibility to irradiation than A549 cells with first cytotoxic effects occurring at 300 J cm^−2^ (405 nm) and 400 J cm^−2^ (450 nm) in comparison to 300 J cm^−2^ and 1000 J cm^−2^, respectively. We could define conditions in which a significant reduction of colony forming units for all ESKAPE pathogens, except *Enterococcus faecium*, was achieved at 405 nm while avoiding cytotoxicity. Irradiation at 450 nm demonstrated a more variable effect which was species and medium dependent. In summary a significant reduction of viable bacteria could be achieved at subtoxic irradiation doses, supporting a potential use of visible light as an antimicrobial agent in clinical settings.

## Introduction

Due to increased numbers of antibiotic resistant bacterial infections in hospital settings^1^, alternative treatment strategies are attracting increased attention in the medical field. Besides bacteriophages^2^, nanoparticles^3^, antimicrobial peptides^4^ and antibiotics in combination with adjuvants^5^, photodynamic therapies and light based approaches are a major focus of current investigations.

Disinfection techniques based on visible light irradiation comprise multiple beneficial characteristics including lower toxicity compared to UV light^6^, no need for the addition of exogenous photosensitizers and a low risk of resistance development^7,8^. Microbial inactivation through visible light is based on photo-excitation of intracellular photosensitizers. These molecules absorb photons of a certain wavelength which induces an oxygen dependent photo-excitation reaction within the bacterial cell^9^. The generated reactive oxygen species subsequently damage multiple intracellular targets^10–14^. Especially violet and blue light irradiation was thoroughly investigated in clinical trials for the treatment of acne vulgaris^15^ and *Helicobacter pylori* stomach infections^16,17^. Furthermore, multiple murine in vivo studies demonstrated the potential of visible light (405–425 nm) for the treatment of wound infections and keratitis^7,18–21^. Visible light of 405 nm was shown to be the most effective wavelength in regard to bacterial inactivation^22,23^, a wavelength correlating with the absorption peak of porphyrins^24,25^. At 450 nm flavins are considered as photosensitizers and lead to a considerable microbial inactivation, although the overall effect is less pronounced than at 405 nm irradiation^23,26–28^.

Besides the antimicrobial effect, cytotoxicity of human cells is a major concern for clinical applications. Similar to the effect in bacterial cells, visible light irradiation can lead to the generation of ROS in human cells^29–31^. However, only a few publications directly compared the effect of visible light irradiation on human and bacterial cells. Dai and coworkers compared the survival of keratinocytes to the inactivation of *Staphylococcus aureus* and *Pseudomonas aeruginosa* in two separate studies with 415 nm irradiation. Both studies showed the presence of a therapeutic window for light application as the keratinocytes survived the irradiation with a dose of 109.9 J cm^−2^ and 170 J cm^−2^, whereas *P. aeruginosa* and *S. aureus* cell densities were reduced by several log levels^18,19^. Similar effects were observed comparing the survival of keratinocytes and *Candida albicans* or *Acinetobacter baumannii*, respectively^7,20^. Concerning 405 nm irradiation, a higher sensitivity of bacterial species was reported in comparison to osteoblasts and fibroblasts^32–34^. Overall, the cytotoxicity in human cells seems to be dose dependent^32,35^ and light in the violet range induces higher toxicity than blue light^36^.

We previously investigated the mechanism of action of microbial photoinactivation and compared the sensitivity of the ESKAPE pathogens and closely related non-pathogenic species against violet and blue light irradiation^13,26^. Here, we report on the potential cytotoxicity of violet and blue light during a prolonged irradiation of human cells that was, to the best of our knowledge, so far not tested. Most current setups of cytotoxicity investigations use human cells that are kept in phosphate buffered saline (PBS) for a short time during the course of the irradiation. In this study cytotoxicity of 405 nm and 450 nm irradiation was determined for monocytic THP-1 cells and the alveolar epithelium cell line A549, in irradiation experiments exceeding 22 h. To be able to compare the effects of visible light on bacterial species and human cells, all experiments were conducted under identical conditions in cell culture medium.

## Methods

### Bacterial strains and cultivation

The bacterial strains used in this study are summarized in Table 1. *Escherichia coli* and *A. baumannii* were incubated in LB-Miller medium (10 g/l tryptone (Gibco, Detroit, MI), 5 g/l yeast extract (BD, Sparks, MD), 10 g/l sodium chloride (AppliChem, Darmstadt, Germany)). The remaining four species were incubated in THY broth (36.4 g/l Todd–Hewitt Broth (Oxoid, Basingstoke, UK), 5 g/l yeast extract (BD, Sparks, MD)).

**Table 1:** Bacterial strains used in the study and their resistance pattern.

Species	Strain	Resistance	Source
*E. coli*	BSU1286*	ESBL	Clinical isolate, Ulm collection
*S. aureus*	ATCC43300	MRSA	ATCC
*K. pneumoniae*	ATCC700603	ESBL	ATCC
*A. baumannii*	ATCC19606	–	ATCC
*P. aeruginosa*	ATCC27853	–	ATCC
*E. faecium*	DSM17050	VRE	DSMZ
*P. aeruginosa*	BSU1295**	–	Clinical isolate, Ulm collection
*P. aeruginosa*	BSU1296	–	Clinical isolate, Ulm collection
*P. aeruginosa*	BSU1297	–	Clinical isolate, Ulm collection
*P. aeruginosa*	BSU1298	–	Clinical isolate, Ulm collection
*S. aureus*	DSM 26309	–	DSMZ
*S. aureus*	ATCC29213	–	ATCC
*S. aureus*	ATCC13565	–	ATCC
*S. aureus*	ATCC25923	–	ATCC

*^26^; **^55^.

### Human cell lines and culture conditions

The monocytic cell line THP-1 and alveolar epithelium cell line A549 were used to quantify the toxicity of the irradiation on human cells. Both cell lines were incubated at 37 °C in a 5% CO_2_ atmosphere in ambient humidified air. THP-1 cells were grown in RPMI 1640 (Gibco™ RPMI 1640 Medium, GlutaMAX™, Life Technologies Limited, Paisley, UK) containing phenol red, supplemented with 0.01 M HEPES Buffer (PAN-Biotech, Aidenbach, Germany), 10% v/v fetal bovine serum (FBS superior stabil, Bio&Sell, Feucht, Germany) and 0.2% v/v 2-mercaptoethanol (SERVA Electrophoresis, Heidelberg, Germany). The A549 cells were incubated in DMEM medium (DMEM, high glucose, Life Technologies Limited, Paisley, UK) containing phenol red, supplemented with 10% v/v fetal bovine serum (FBS superior stabil, Bio&Sell, Feucht, Germany) and 1 mM sodium pyruvate (Life Technologies Limited, Paisley, UK). Both cell culture media were supplemented with 1% v/v Penicillin–Streptomycin (PAN-Biotech, Aidenbach, Germany).

### Irradiation setup

Two different irradiation setups were tested, one with a peak wavelength of 405 nm (Nichia NVSU233B SMD-LED, Nichia corp., Anan, Japan) and one in the blue range at 450 nm (Cree XP-E2 SMD-LED XPEBRY-L1-0000-00N01,Cree Inc., Durham, NC). Six LEDs each were incorporated in a highly reflective hollow pyramid as described before^26^. An intensity of 20 mW cm^−2^ was applied and verified with an optical power meter OPM150 (Qioptiq, Göttingen, Germany). The pyramid was placed on top of a 24-well plate containing the samples and the whole setup was incorporated in an incubator ensuring a constant temperature of 37 °C and a 5% CO_2_ atmosphere in ambient air.

### Bacterial irradiation

*E. coli* and *A. baumannii* were incubated at 37 °C on a rotary shaker and the remaining strains were cultured at 37 °C in the presence of 5% CO_2_ in ambient air in an incubator. Mid-exponentially grown bacteria were harvested by centrifugation at 13,000*g* for 1 min. The bacterial pellet was washed once in either RPMI or DMEM medium without antibiotics followed by dilution to the desired density of 5 × 10^7^ colony forming units per ml (cfu ml^−1^). 300 µl of the bacterial suspensions were added into each well of a 24-well plate. A separate plate was set up which served as negative control to check for the growth of the non-irradiated bacteria. The cfu ml^−1^ was determined for each well in the beginning of the experiment by plating dilutions of the bacterial suspension on sheep blood agar plates (TSA + SB, Oxoid, Basingstoke, UK). The sample plate was placed together with the irradiation pyramid into the incubator and samples were drawn in certain time intervals to quantify the surviving bacteria by plating dilutions on sheep blood agar plates. After incubation for 24 h at 37 °C in a 5% CO_2_ atmosphere, the bacteria were enumerated and the survival of the bacteria was calculated in comparison to the starting cfu ml^−1^. For each experiment three biological replicates with each three technical replicates were performed.

### Toxicity measurement

The LDH Cytotoxicity Detection Kit (Takara Bio, Kusatsu, Japan) was used to measure the survival of irradiated human cells. The lactate dehydrogenase is a stable enzyme that is released from human cells upon plasma membrane damage and thus the enzyme activity in the cell free supernatant reflects the amount of damaged cells. The assay was performed according to the instruction of the manufacturer. Briefly, 1 ml of THP-1 cells with a density 1 × 10^6^ cells ml^−1^ was seeded into the wells of two 24-well plates. One plate served as negative control and was incubated in a metal box in the same incubator as the irradiated plate. At certain time intervals the samples were collected and centrifuged at 400*g* for 5 min followed by the measurement of the LDH activity in the supernatant. As positive control TritonX-100 (1% in RPMI medium) was used and RPMI medium without cells served as negative control. For the determination of the survival of A549 cells, 1 ml of 8 × 10^4^ cells ml^−1^ were seeded into 24-well plates. After an incubation of 24 h at 37 °C in a 5% CO_2_ atmosphere, one plate was transferred into the irradiation setup and the negative control plate was placed into a metal box in the same incubator. At selected time intervals, 500 µl of the medium was discarded and 500 µl of fresh DMEM medium or DMEM medium containing 2% TritonX-100 (final concentration 1%) was added to each sample. The samples were collected and centrifuged for 5 min at 400*g* followed by the determination of the LDH activity according to the instructions of the manufacturer. The cytotoxicity is depicted as % of the positive control and each time point was repeated in three biological experiments with three technical replicates each. A cytotoxic effect is proposed for samples exceeding 10% dead cells, the highest amount measured in control samples.

### THP-1 infection assay

THP-1 cells were seeded in a density of 10^6^ cells per well in 6-well tissue culture plates and were incubated for 24 h in the presence of 50 ng ml^−1^ Phorbol-12-myristat-13-acetat (PMA) to generate adherent macrophages, as previously described^37^. For the infection procedure the THP-1 cells were washed two times with RPMI 1640 medium without antibiotics. Mid-exponentially grown bacteria were harvested by centrifugation and washed once in RPMI medium without antibiotics. 10^6^ bacterial cells were incubated together with 10^6^ stimulated THP-1 cells (multiplicity of infection [MOI] 1:1) for 1 h at 37 °C in the presence of 5% CO_2_ in ambient air. Afterwards the plate was either irradiated with 405 nm light with a dose of 25 J cm^−2^ or incubated in the dark. The medium was collected and the stimulated THP-1 cells were lysed by the addition of 1% Triton-X 100 in ddH_2_O. As growth control, bacteria were incubated in the absence of stimulated THP-1 cells for the course of the experiment. To be able to enumerate the total bacterial number that survived the procedure, dilutions of the mixture were plated on sheep blood agar plates and incubated for 24 h at 37 °C in a 5% CO_2_ atmosphere. The results are illustrated as percent survival of the respective sample in comparison to its not irradiated control.

### Statistical analysis

Statistical significance was tested using the Welch two-sample *t* test on log-transformed data. The hypothesized mean difference was assumed to be zero. *P* values of less than 0.05 were considered statistically significant.

## Results

### Irradiation effect on human cell lines

**Figure 1:**
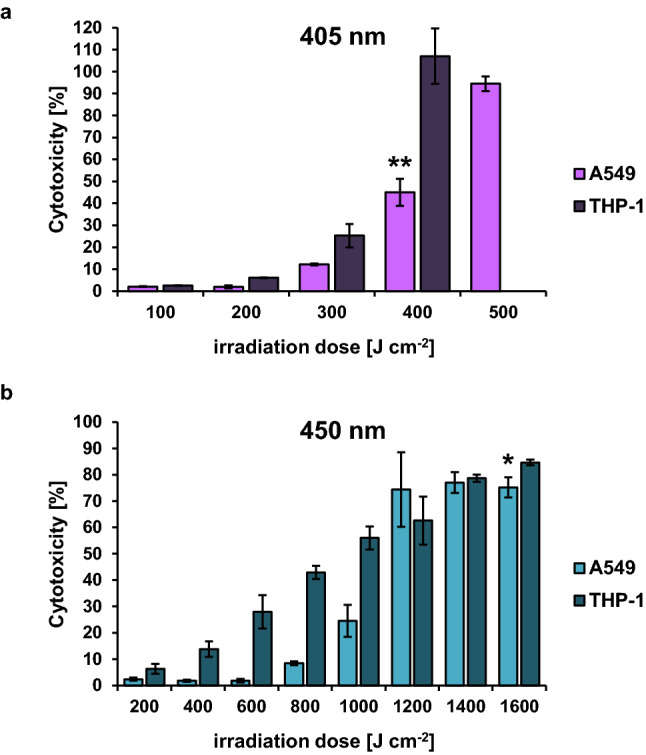
Light induced cytotoxicity in the human cell lines THP-1 and A549 measured by LDH activity. (**a**) Irradiation of cells at a wavelength of 405 nm. (**b**) Irradiation of cells at a wavelength of 450 nm. The values are expressed as % cytotoxicity of the positive control (TritonX-100 treated cells). The mean and standard deviation of three independent biological replicates with each three technical replicates are illustrated. The asterisk indicates statistically significant difference between the two cell lines (*p < 0.05; **p < 0.01).

To assess the immediate cytotoxicity of blue and violet light irradiation on THP-1 and A549 cells, LDH activity in culture supernatant was measured (Fig. 1). Cells incubated in the dark served as negative control and resulted in around 10% dead cells after 22 h incubation for THP-1 and less than 5% for A549 cells (Fig. S1). With 405 nm irradiation first cytotoxic effects could be observed at a dose of 300 J cm^−2^ for THP-1 and A549 cells. Higher doses were needed to elicit cytotoxicity at 450 nm irradiation. First cytotoxic effects were observed at 400 J cm^−2^ for THP-1 cells whereas 1000 J cm^−2^ was needed to trigger cell death in A549 cells.

### Irradiation of ESKAPE pathogens in presence of cell culture media

**Figure 2:**
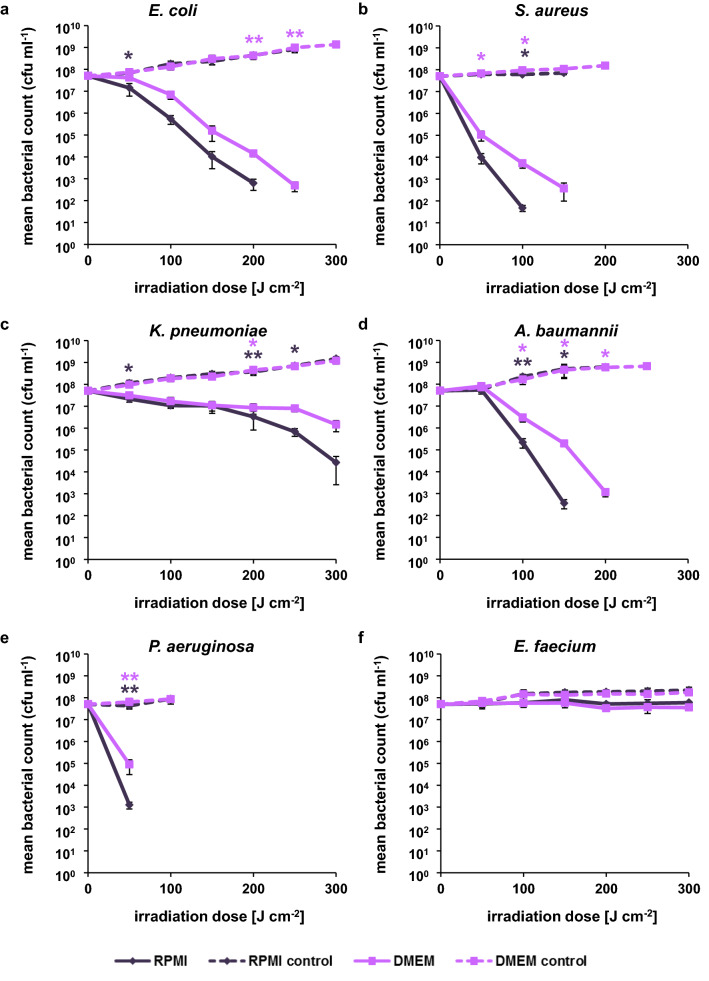
Survival of ESKAPE pathogens in the presence of RPMI and DMEM medium irradiated with 405 nm. (**a)** *E. coli*. (**b**) *S. aureus* (ATCC43300). (**c**) *K. pneumoniae*. (**d**) *A. baumannii*. (**e**) *P. aeruginosa* (ATCC27853). (**f**) *E. faecium*. The values are expressed as log survival in comparison to starting bacterial density of 5 × 10^7^ cfu ml^−1^. The mean and standard deviation of three independent biological replicates with each three technical replicates are illustrated. Statistically significant differences between the irradiated samples and their respective controls are indicated with asterisks in the respective colors (*p < 0.05; **p < 0.01).

To be able to compare the effect of visible light on bacteria and human cells, the disinfection capacity of blue and violet light irradiation was tested against ESKAPE pathogens incubated in cell culture medium. At 405 nm irradiation all species except *Enterococcus faecium* showed reduced survival at a dose of up to 300 J cm^−2^ (Fig. 2). For all affected species the inactivation in RPMI medium was more pronounced than in DMEM medium. Complete sterility of the samples could be achieved for *E. coli*, *S. aureus*, *A. baumannii* and *P. aeruginosa* at a dose of 250 J cm^−2^, 150 J cm^−2^, 200 J cm^−2^ and 100 J cm^−2^ in RPMI medium and a dose of 300 J cm^−2^, 200 J cm^−2^, 250 J cm^−2^ and 100 J cm^−2^ in DMEM medium, respectively. The irradiated *E. faecium* sample showed no inactivation whereas the corresponding control showed moderate growth. For *Klebsiella pneumoniae*, a reduction of 3.5 log could be achieved in the presence of RPMI medium and a reduction of almost 2 log in the presence of DMEM medium.

**Figure 3:**
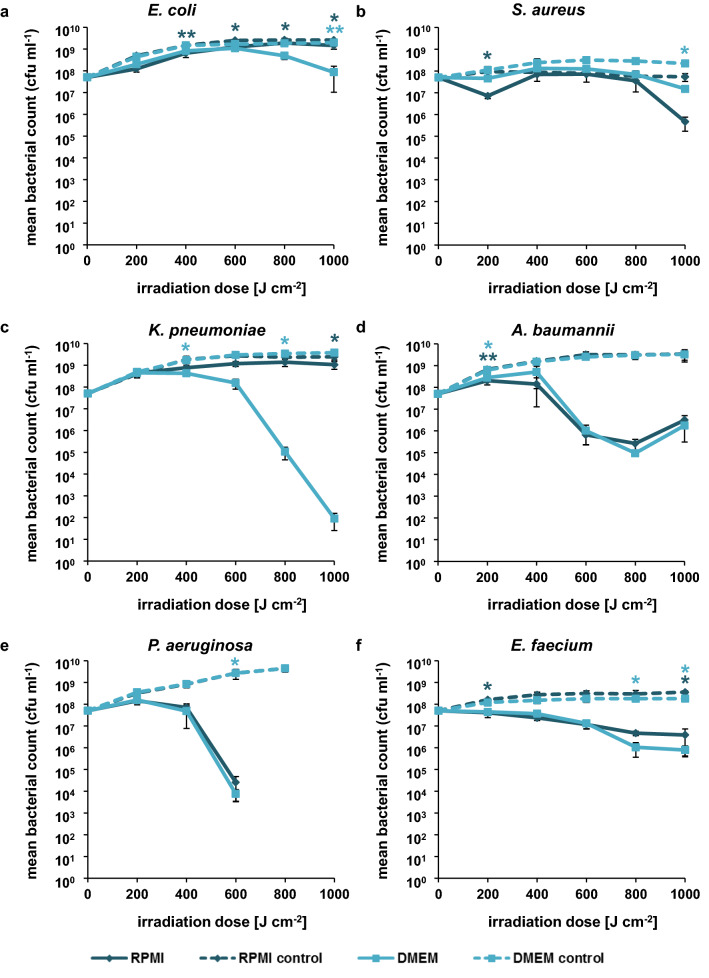
Survival of ESKAPE pathogens in the presence of RPMI and DMEM medium irradiated with 450 nm. (**a)** *E. coli*. (**b**) *S. aureus* (ATCC43300). (**c**) *K. pneumoniae*. (**d**) *A. baumannii*. (**e**) *P. aeruginosa* (ATCC27853). (**f**) *E. faecium*. The values are expressed as log survival in comparison to starting bacterial density of 5 × 10^7^ cfu ml^−1^. The mean and standard deviation of three independent biological replicates with each three technical replicates are illustrated. Statistically significant differences between the irradiated samples and their respective controls are indicated with asterisks in the respective colors (*p < 0.05; **p < 0.01).

In contrast, the irradiation at 450 nm resulted only in a complete inactivation of *P. aeruginosa* during the course of the experiment (Fig. 3). A 2 log reduction in bacterial number could be achieved for *A. baumannii* at 800 J cm^−2^ before a regrowth could be observed. *S. aureus* was only moderately affected by 450 nm light irradiation and no difference could be observed between irradiated *E. coli* samples grown in RPMI medium and their respective control, whereas *E. coli* cells in DMEM medium showed a reduction of cell density for 1000 J cm^−2^. For *K. pneumoniae* a pronounced difference could be observed between RPMI incubated bacteria and DMEM incubated bacteria. The sample irradiated in the presence of RPMI showed a moderate growth during the course of the experiment although weaker than the corresponding dark control. The DMEM sample however showed a reduction of almost 6 log at a dose of 1000 J cm^−2^. Both irradiated *E. faecium* samples behaved similarly with a moderate reduction of cell density up to a dose of 1000 J cm^−2^.

To test the killing potential of violet and blue light irradiation on different bacterial strains, additional *P. aeruginosa* (Gram negative, Supplementary Table S1) and *S. aureus* (Gram positive, Supplementary Table S2) strains were investigated. The *S. aureus* strains showed a comparable killing to the previously investigated ATCC43300 strain at 405 and 450 nm. While comparable killing was observed at 405 nm for the *P. aeruginosa* strains, the log reduction values were diminished at 450 nm.

### THP-1 infection assay

**Figure 4:**
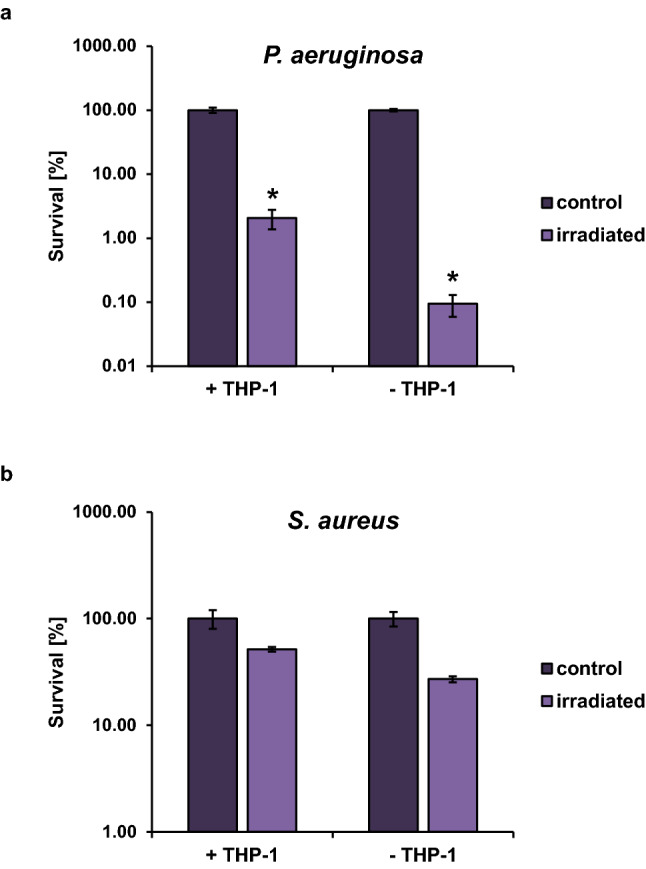
THP-1 infection assay. PMA-stimulated THP-1 cells were infected at a MOI of 1. (**a**) *P. aeruginosa* (ATCC27853). (**b**) *S. aureus* (ATCC43300). Incubation of the bacteria without cells served as control. After 1 h incubation, the samples were either irradiated with 405 nm light with a dose of 25 J cm^−2^ or kept in the dark. Values represent survival of the irradiated samples in comparison to the dark control. The mean and standard deviation of three independent biological replicates with each three technical replicates are illustrated. Statistically significant differences between the irradiated samples and their respective controls are indicated with an asterisk (*p < 0.05).

To investigate the effect of 405 nm irradiation on the survival of *P. aeruginosa* and *S. aureus* in an infection model, THP-1 monocytic cells were stimulated with PMA to generate a macrophage like cell line. After 1 h preincubation of the cells with the bacteria, the respective samples were irradiated (25 J cm^−2^) and the total number of surviving bacteria was assessed. Also in the presence of eukaryotic cells, the bacteria are susceptible to 405 nm irradiation (Fig. 4). For both bacterial species, the killing efficiency of the violet light decreased the number of surviving bacteria in infection experiments compared to the non-irradiated samples. While bacterial killing was even more efficient in samples without eukaryotic cells, this is not possible in an infection situation, where host cells will always be present. The PMA stimulated THP-1 cells showed no loss in viability for the applied irradiation doses (Supplementary Fig. S2).

## Discussion

Hospital acquired infections are associated with increased patient mortality and excess costs for the healthcare sector^38^. The majority of these infections are caused by bacterial species of the ESKAPE group with a high incidence of antibiotic resistant isolates^39,40^. To improve this situation the development of alternative treatment strategies is crucial.

Of particular interest are intervention strategies based on the antimicrobial capacity of visible light as no further adjuvants are needed. Several in vivo studies of visible light therapy in murine infection models and first clinical studies propose a therapeutic window in which pathogens are inactivated without damage to host cells^18–20,32,41,42^. However, there is still a lack of studies investigating the cellular effect of irradiation over a longer timeframe. In addition, the antimicrobial activity of visible light irradiation and the potential cytotoxicity in human cells are rarely measured in the same experimental setup. Therefore, we compared the survival of ESKAPE pathogens with the effect of blue and violet light irradiation on human cells under identical experimental conditions.

Apart from one study that measured the effect of blue light on dendritic cells incubated in RPMI medium using a dose of up to 15 J cm^−2^^43^, investigations of cytotoxicity so far were mainly conducted with human cells incubated in PBS. As our experimental design necessitates an irradiation of up to 22 h (1600 J cm^−2^), the human cells had to be incubated in classical cell culture medium to ensure survival. As previously reported, cytotoxic effects in human cells were shown to be wavelength dependent, with shorter wavelengths being less tolerable^36^. First cytotoxic effects in monocytic THP-1 cells were detectable at 300 J cm^−2^ for 405 nm irradiation and 400 J cm^−2^ for 450 nm irradiation. A similar pattern could be observed for the alveolar epithelium cell line A549 with 300 J cm^−2^ and 1000 J cm^−2^, respectively.

Similar to the cytotoxicity measurements in human cells, the majority of light induced inactivation experiments with bacteria were conducted in PBS. To be able to compare the effect of visible light on human cells and pathogens we performed the antimicrobial experiments in RPMI (cell culture media of THP-1 cells) and DMEM (A549 cells) medium. With 405 nm irradiation a complete inactivation of *E. coli*, *S. aureus*, *A. baumannii* and *P. aeruginosa* could be achieved with a dose of up to 300 J cm^−2^, irrespective of the medium composition. In contrast, only a slight decrease in comparison to the control could be detected for *E. faecium* and a moderate reduction in the mean bacterial count for *K. pneumoniae* (Fig. 2). The irradiation dose necessary to reduce the bacterial density thereby differed substantially from our previous determination of inactivation of the same ESKAPE pathogens incubated in PBS (Table 2)^26^. At 405 nm the antimicrobial effect detected in cell culture medium exceeded in all species, except for *E. faecium*, the results obtained in PBS. In case of 450 nm irradiation the inactivation seemed to be species and medium dependent. Whereas the effects observed for *A. baumannii* and *P. aeruginosa* were comparable between RPMI and DMEM medium, bacteria in PBS showed a higher sensitivity, a pattern we also observed for *S. aureus* (Table 3). A major difference between the inactivation in RPMI and DMEM could be observed for *K. pneumoniae* with only a slight reduction of the mean bacterial count for RPMI samples and an almost 6 log reduction for bacteria in DMEM medium irradiated at a dose of 1000 J cm^−2^. The values obtained for *K. pneumoniae* in DMEM showed a higher reduction of bacterial counts than experiments conducted in PBS. *E. coli* and *E. faecium* proved to be quiet resistant to 450 nm irrespective of the medium composition. One explanation for the low susceptibility of enterococci against violet and blue light irradiation is the lack of porphyrin synthesis in these species. However, other molecules like NADH, flavins and their photodegradation products could serve as photosensitizers, which may explain the lack of growth of *E. faecium* in the irradiated samples (Figs. 2f, 3f)^44^.

**Table 2:** Irradiation dose [J cm^−2^] per log reduction at 405 nm in comparison to the dark control.

	PBS*			RPMI			DMEM		
	1	2	3	1	2	3	1	2	3
*E. coli*	525	625	725	100	100	150	100	150	150
*S. aureus*	45	135	180	50	50	50	50	50	100
*K. pneumoniae*	525	725	725	100	200	250	100	300	–
*A. baumannii*	90	90	135	100	100	100	100	150	150
*P. aeruginosa*	90	135	180	50	50	50	50	50	100
*E. faecium*	525	725	–	–	–	–	–	–	–

*Values derived from^26^.

**Table 3:** Irradiation dose [J cm^−2^] per log reduction at 450 nm in comparison to the dark control.

	PBS*			RPMI			DMEM		
	1	2	3	1	2	3	1	2	3
*E. coli*	1750	2250	2500	–	–	–	1000	–	–
*S. aureus*	160	240	320	1000	1000	–	1000	–	–
*K. pneumoniae*	1000	1500	–	–	–	–	600	800	800
*A. baumannii*	90	90	135	400	600	600	600	600	600
*P. aeruginosa*	90	135	180	400	600	600	400	600	600
*E. faecium*	750	1000	1250	400	–	–	600	800	–

*Values derived from^26^.

To investigate if the observed bacterial killing during light irradiation is strain specific, we tested different *P. aeruginosa* and *S. aureus* strains as model organisms for Gram negative and Gram positive species. While the killing efficiency of 405 nm irradiation was comparable between the strains, *P. aeruginosa* isolates showed diminished reduction values at 450 nm irradiation (Supplementary Table S1). A variability of the necessary irradiation dose that is needed to trigger bacterial killing in strains of the same species is already reported but so far mainly attributed to different experimental setups in different laboratories^23^. The variability of the *P. aeruginosa* isolates towards killing at 450 nm irradiation therefore emphasizes further investigations of multiple strains in the same irradiation setup to better understand the differences in susceptibility of clinical isolates of the same species towards visible light irradiation.

The simultaneous irradiation of bacteria and human cells in an infection assay further proved that bacteria are killed by violet light irradiation at an irradiation dose which does not impede the survival of the human cells, in this case PMA stimulated THP-1 cells. The human cells appeared to protect against the killing of the bacteria during irradiation to a certain extent, as bacteria in absence of the cells were killed more efficiently.

The irradiation in cell culture medium differs substantially in two main aspects from the irradiation in PBS. The presence of nutrients in cell culture medium provides energy for the bacteria to grow and to repair the damage caused by ROS. The impact of available nutrients during irradiation was previously investigated by Vollmerhausen et al*.* for uropathogenic *E. coli* grown in urine mucin media^45^. The study demonstrated that the dissolved oxygen levels were reduced in *E. coli* suspension in urine mucin media in comparison to PBS leading to a decreased disinfection capacity of visible light, an observation that could explain the effects of blue light irradiation on *A. baumannii* (Fig. 3d). After an initial decrease in the mean bacterial count (up to 800 J cm^−2^) the bacterial number increases again, indicating that a reduced oxygen level may impede the generation of ROS.

Nutrient rich cell culture media contain possible photosensitizer, like riboflavin often in combination with tryptophan, tyrosine, phenol red and HEPES^46–49^. Thus, the increased antimicrobial effect of irradiation at 405 nm in cell culture media in comparison to PBS was expected. Surprisingly irradiation at 450 nm in cell culture medium was inferior to irradiation of the samples in PBS. One possible explanation is that irradiation with blue light kills only a part of the population in comparison to violet light and the presence of nutrients in cell culture media allows regrowth of the remaining bacterial population.

A possibility to reduce the effect of the photosensitizers in cell culture medium in our experimental setup would be the use of cell culture media designed for live cell imaging. These photo-inert media contain antioxidant rich supplements and are used in specific applications like optogenetics^50^. The drawback of this alternative is the unknown relevance of such media compositions for in vivo applications which is also true for the classical cell culture media that were designed to mimic the extracellular milieu but can only partially reflect in vivo redox settings^51^. Additionally cell cultures are routinely incubated in 95% air and 5% CO_2_, an oxygen tension which exceeds the one usually encountered by most cells in a living organism and which facilitates the production of ROS^52^.

This study highlights the implications of the experimental setup on the antimicrobial activity of visible light irradiation and the potential phototoxic effects on human cells. Nevertheless, due to the above discussed implications, the irradiation of human cells in cell culture medium, as required for long term irradiation experiments, is of limited use to predict a safe irradiation dose for clinical applications. Without doubt, irradiation with visible light is a promising tool as it is able to inactivate various bacterial and fungal pathogens which is already reflected by the potential therapeutic use of visible light^53^. For these purposes, such as the development of endotracheal tubes with incorporated blue light emitting diodes (LEDs)^54^, phototoxicity of visible light irradiation can only be poorly predicted in vitro and needs further evaluation in in vivo experiments.

## Supplementary Information

Supplementary Information.

## Data Availability

The datasets generated during and/or analyzed during the current study are available from the corresponding author on reasonable request.

## References

[1] Boucher HW (2009). Bad bugs, no drugs: No eskape! An update from the infectious diseases society of America. Clin. Infect. Dis..

[2] Domingo-Calap, P. & Delgado-Martinez, J. Bacteriophages: Protagonists of a post-antibiotic era. *Antibiotics (Basel)*. 10.3390/antibiotics7030066 (2018). 10.3390/antibiotics7030066 PMC6163168 30060506

[3] Beyth N, Houri-Haddad Y, Domb A, Khan W, Hazan R (2015). Alternative antimicrobial approach: Nano-antimicrobial materials. Evid. Based Complement. Alternat. Med..

[4] Gomes B (2018). Designing improved active peptides for therapeutic approaches against infectious diseases. Biotechnol. Adv..

[5] Gonzalez-Bello C (2017). Antibiotic adjuvants—A strategy to unlock bacterial resistance to antibiotics. Bioorg. Med. Chem. Lett..

[6] Kleinpenning MM (2010). Clinical and histological effects of blue light on normal skin. Photodermatol. Photoimmunol. Photomed..

[7] Zhang Y (2014). Antimicrobial blue light therapy for multidrug-resistant acinetobacter baumannii infection in a mouse burn model: Implications for prophylaxis and treatment of combat-related wound infections. J. Infect. Dis..

[8] Amin RM, Bhayana B, Hamblin MR, Dai T (2016). Antimicrobial blue light inactivation of pseudomonas aeruginosa by photo-excitation of endogenous porphyrins: In vitro and in vivo studies. Lasers Surg. Med..

[9] Hamblin MR, Abrahamse H (2019). Can light-based approaches overcome antimicrobial resistance?. Drug Dev. Res..

[10] Adair TL, Drum BE (2016). Rna-seq reveals changes in the *Staphylococcus aureus* transcriptome following blue light illumination. Genom Data.

[11] Kim, M. J. & Yuk, H. G. Antibacterial mechanism of 405-nanometer light-emitting diode against salmonella at refrigeration temperature. *Appl. Environ. Microbiol*. 10.1128/AEM.02582-16 (2017). 10.1128/AEM.02582-16 PMC5311417 28003197

[12] Djouiai, B. *et al.* Role of DNA repair and protective components in bacillus subtilis spore resistance to inactivation by 400-nm-wavelength blue light. *Appl. Environ. Microbiol.*10.1128/AEM.01604-18 (2018). 10.1128/AEM.01604-18 PMC6147000 30054368

[13] Hoenes, K., Bauer, R., Spellerberg, B. & Hessling, M. Microbial photoinactivation by visible light results in limited loss of membrane integrity. *Antibiotics (Basel)*. 10.3390/antibiotics10030341 (2021). 10.3390/antibiotics10030341 PMC8005082 33807025

[14] Chu Z (2019). Inactivation of cronobacter sakazakii by blue light illumination and the resulting oxidative damage to fatty acids. Can. J. Microbiol..

[15] Pei S, Inamadar AC, Adya KA, Tsoukas MM (2015). Light-based therapies in acne treatment. Indian Dermatol. Online J..

[16] Ganz RA (2005). *Helicobacter pylori* in patients can be killed by visible light. Lasers Surg. Med..

[17] Lembo AJ (2009). Treatment of *Helicobacter pylori* infection with intra-gastric violet light phototherapy: A pilot clinical trial. Lasers Surg. Med..

[18] Dai T (2013). Blue light eliminates community-acquired methicillin-resistant *Staphylococcus aureus* in infected mouse skin abrasions. Photomed. Laser Surg..

[19] Dai T (2013). Blue light rescues mice from potentially fatal *Pseudomonas aeruginosa* burn infection: Efficacy, safety, and mechanism of action. Antimicrob. Agents Chemother..

[20] Zhang Y (2016). Antimicrobial blue light inactivation of candida albicans: In vitro and in vivo studies. Virulence.

[21] Zhu H (2017). Antimicrobial blue light therapy for infectious keratitis: Ex vivo and in vivo studies. Invest. Ophthalmol. Vis. Sci..

[22] Maclean M, MacGregor SJ, Anderson JG, Woolsey G (2008). High-intensity narrow-spectrum light inactivation and wavelength sensitivity of *Staphylococcus aureus*. FEMS Microbiol. Lett..

[23] Hessling, M., Spellerberg, B. & Hoenes, K. Photoinactivation of bacteria by endogenous photosensitizers and exposure to visible light of different wavelengths—A review on existing data. *FEMS Microbiol. Lett.*10.1093/femsle/fnw270 (2017). 10.1093/femsle/fnw270 27915252

[24] Ashkenazi H, Malik Z, Harth Y, Nitzan Y (2003). Eradication of propionibacterium acnes by its endogenic porphyrins after illumination with high intensity blue light. FEMS Immunol. Med. Microbiol..

[25] Hamblin MR (2005). *Helicobacter pylori* accumulates photoactive porphyrins and is killed by visible light. Antimicrob. Agents Chemother..

[26] Hoenes K, Bauer R, Meurle T, Spellerberg B, Hessling M (2020). Inactivation effect of violet and blue light on eskape pathogens and closely related non-pathogenic bacterial species—A promising tool against antibiotic-sensitive and antibiotic-resistant microorganisms. Front. Microbiol..

[27] Cieplik F (2014). Blue light kills aggregatibacter actinomycetemcomitans due to its endogenous photosensitizers. Clin. Oral. Investig..

[28] Plavskii VY (2018). Porphyrins and flavins as endogenous acceptors of optical radiation of blue spectral region determining photoinactivation of microbial cells. J. Photochem. Photobiol. B.

[29] Yoshida A (2015). Blue light irradiation-induced oxidative stress in vivo via ros generation in rat gingival tissue. J. Photochem. Photobiol. B.

[30] Vandersee S, Beyer M, Lademann J, Darvin ME (2015). Blue-violet light irradiation dose dependently decreases carotenoids in human skin, which indicates the generation of free radicals. Oxid. Med. Cell Longev..

[31] Del Olmo-Aguado S, Nunez-Alvarez C, Osborne NN (2016). Blue light action on mitochondria leads to cell death by necroptosis. Neurochem. Res..

[32] Ramakrishnan P, Maclean M, MacGregor SJ, Anderson JG, Grant MH (2014). Differential sensitivity of osteoblasts and bacterial pathogens to 405-nm light highlighting potential for decontamination applications in orthopedic surgery. J. Biomed. Opt..

[33] McDonald RS (2013). 405 nm light exposure of osteoblasts and inactivation of bacterial isolates from arthroplasty patients: Potential for new disinfection applications?. Eur. Cell Mater..

[34] McDonald R, Macgregor SJ, Anderson JG, Maclean M, Grant MH (2011). Effect of 405-nm high-intensity narrow-spectrum light on fibroblast-populated collagen lattices: An in vitro model of wound healing. J. Biomed. Opt..

[35] Masson-Meyers DS, Bumah VV, Enwemeka CS (2016). A comparison of four methods for determining viability in human dermal fibroblasts irradiated with blue light. J. Pharmacol. Toxicol. Methods.

[36] Oplander C (2011). Effects of blue light irradiation on human dermal fibroblasts. J. Photochem. Photobiol. B.

[37] Sagar A (2013). The beta-hemolysin and intracellular survival of streptococcus agalactiae in human macrophages. PLoS ONE.

[38] Umscheid CA (2011). Estimating the proportion of healthcare-associated infections that are reasonably preventable and the related mortality and costs. Infect. Control Hosp. Epidemiol..

[39] Rossolini GM, Arena F, Pecile P, Pollini S (2014). Update on the antibiotic resistance crisis. Curr. Opin. Pharmacol..

[40] Pendleton JN, Gorman SP, Gilmore BF (2013). Clinical relevance of the eskape pathogens. Expert Rev. Anti Infect. Ther..

[41] Soukos NS, Stultz J, Abernethy AD, Goodson JM (2015). Phototargeting human periodontal pathogens in vivo. Lasers Med. Sci..

[42] Genina EA, Titorenko VA, Belikov AV, Bashkatov AN, Tuchin VV (2015). Adjunctive dental therapy via tooth plaque reduction and gingivitis treatment by blue light-emitting diodes tooth brushing. J. Biomed. Opt..

[43] Fischer MR (2013). Blue light irradiation suppresses dendritic cells activation in vitro. Exp. Dermatol..

[44] Hessling M, Wenzel U, Meurle T, Spellerberg B, Hones K (2020). Photoinactivation results of enterococcus moraviensis with blue and violet light suggest the involvement of an unconsidered photosensitizer. Biochem. Biophys. Res. Commun..

[45] Vollmerhausen TL (2017). Visible and UV light as a potential means of preventing *Escherichia coli* biofilm formation in urine and on materials used in urethral catheters. J. Photochem. Photobiol. B.

[46] Yanuk JG (2017). A comprehensive analysis of direct and photosensitized attenuation of *Toxoplasma**gondii* tachyzoites. J. Photochem. Photobiol. B.

[47] Zigler JS, Lepe-Zuniga JL, Vistica B, Gery I (1985). Analysis of the cytotoxic effects of light-exposed hepes-containing culture medium. In Vitro Cell Dev. Biol..

[48] Stoien, J. D. & Wang, R. J. Effect of near-ultraviolet and visible light on mammalian cells in culture ii. Formation of toxic photoproducts in tissue culture medium by blacklight. *Proc. Natl. Acad. Sci. USA* **71**, 3961–3965. 10.1073/pnas.71.10.3961 (1974). 10.1073/pnas.71.10.3961 PMC434306 4530275

[49] Grzelak A, Rychlik B, Bartosz G (2001). Light-dependent generation of reactive oxygen species in cell culture media. Free Radic. Biol. Med..

[50] Stockley JH (2017). Surpassing light-induced cell damage in vitro with novel cell culture media. Sci. Rep..

[51] Spasojevic I (2016). What if cell culture media do not mimic in vivo redox settings?. Redox Rep..

[52] Halliwell B (2014). Cell culture, oxidative stress, and antioxidants: Avoiding pitfalls. Biomed. J..

[53] Wang Y (2017). Antimicrobial blue light inactivation of pathogenic microbes: State of the art. Drug Resist. Updat..

[54] Sicks B, Hoenes K, Spellerberg B, Hessling M (2020). Blue leds in endotracheal tubes may prevent ventilator-associated pneumonia. Photobiomodul. Photomed. Laser Surg..

[55] Gross R (2020). A placenta derived c-terminal fragment of beta-hemoglobin with combined antibacterial and antiviral activity. Front. Microbiol..

