# Supplementary material for: The effects of violet and blue light irradiation on ESKAPE pathogens and human cells in presence of cell culture media
Source: Sci Rep. 2021 Dec 28;11:24473. doi: 10.1038/s41598-021-04202-x (PMC8714816; doi:10.1038/s41598-021-04202-x)
Supplement: Supplementary file 1 — Supplementary Information. [file 41598_2021_4202_MOESM1_ESM.pdf]

Figure S1

a

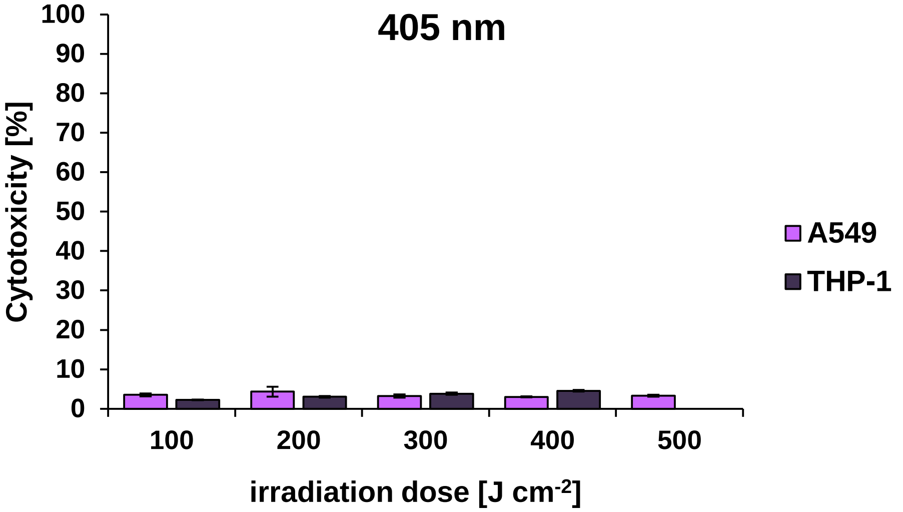

b

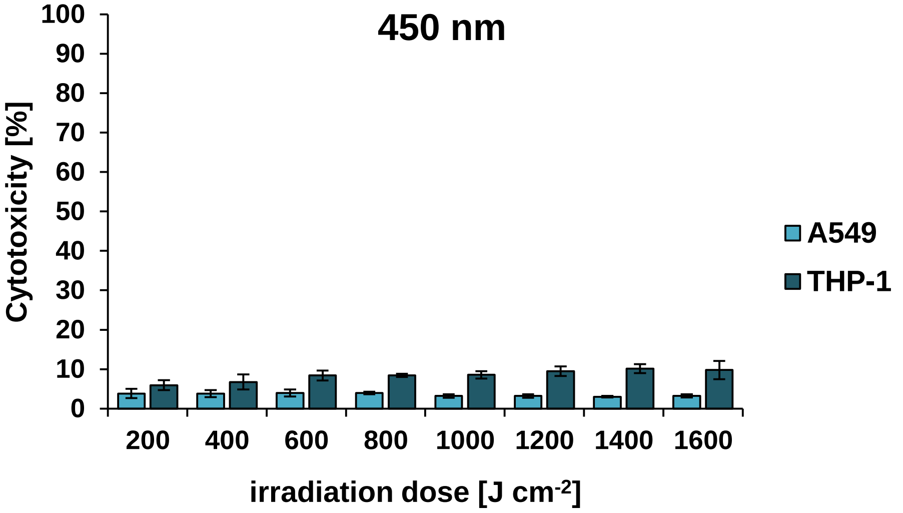

Figure S1: Percentage of dead cells in negative control samples incubated in the dark measured by LDH activity. To be able to compare the values to the irradiated samples (Figure 1), the irradiation dose is illustrated instead of the time although the control samples were not irradiated; e.g. 100 J cm^{-2} (irradiation intensity of 20 mW cm^{-2}) corresponds to 1 h 23 min 20 s incubation in the dark. **a**: Control samples for the irradiation at a wavelength of 405 nm. **b**: Control samples for the irradiation at a wavelength of 450 nm. The values are expressed as % cytotoxicity of the positive control (TritonX-100 treated cells). The mean and standard deviation of three independent biological replicates with each three technical replicates are illustrated.

Figure S2

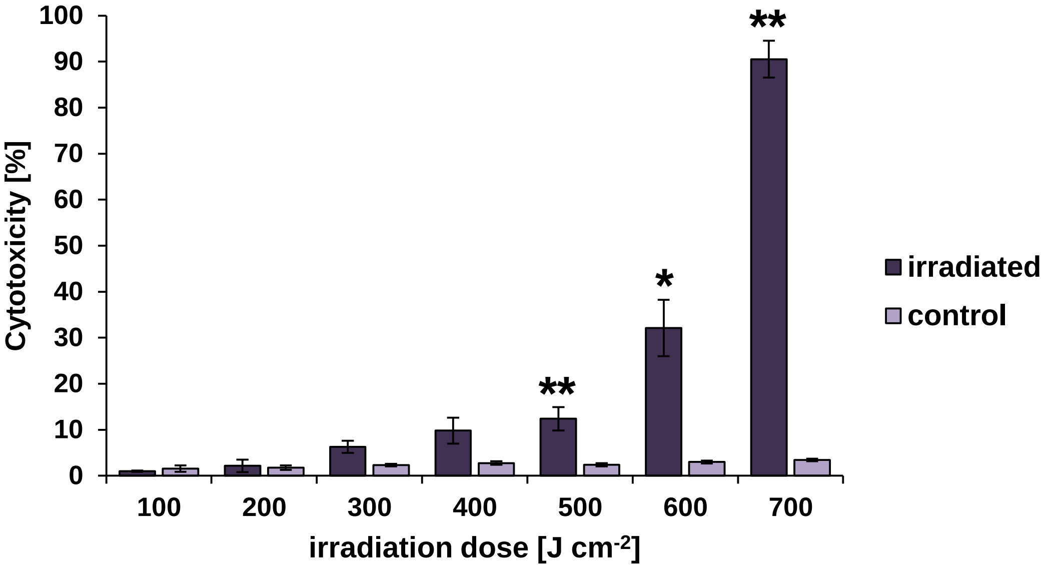

Figure S2: Light induced cytotoxicity in the PMA stimulated THP-1 cell line measured by LDH activity. The cells were irradiated with 405 nm light. The values are expressed as % cytotoxicity of the positive control (TritonX-100 treated cells). The mean and standard deviation of three independent biological replicates with each three technical replicates are illustrated. The asterisk indicates statistically significant difference between the irradiated sample and the respective control (*$p < 0.05$; **$p < 0.01$).

Table S1

		ATCC27853	BSU1295	BSU1296	BSU1297	BSU1298
**405 nm**	RPMI	4.54±0.03	5.23±0.24	7.35±0.42	5.53±0.42	7.28±0.07
	DMEM	2.94±0.16	3.71±0.10	2.85±0.17	3.19±0.35	4.07±0.21
**450 nm**	RPMI	5.16±0.48	0.05±0.02	0.31±0.12	0.37±0.13	1.86±0.15
	DMEM	5.57±0.05	0.37±0.10	0.46±0.10	0.48±0.13	2.14±0.12

Table S1: Survival of *P. aeruginosa* strains in the presence of RPMI and DMEM medium irradiated with 405 nm (dose 50 J cm^{-2}) and 450 nm (dose 600 J cm^{-2}). The values are expressed as log reduction in comparison to the dark control. The mean and standard deviation of three independent biological replicates with each three technical replicates are illustrated.

Table S2

		ATCC43300	DSM 26309	ATCC29213	ATCC13565	ATCC25923
**405 nm**	RPMI	3.84±0.26	2.12±0.11	2.94±0.42	4.05±0.21	2.60±0.24
	DMEM	2.86±0.20	2.04±0.16	2.03±0.12	2.52±0.09	1.84±0.20
**450 nm**	RPMI	2.15±0.39	1.24±0.14	1.61±0.08	3.02±0.27	0.56±0.09
	DMEM	1.17±0.21	0.66±0.2	1.02±0.17	2.14±0.12	0.72±0.09

Table S2: Survival of *S. aureus* strains in the presence of RPMI and DMEM medium irradiated with 405 nm (dose 50 J cm^{-2}) and 450 nm (dose 1000 J cm^{-2}). The values are expressed as log reduction in comparison to the dark control. The mean and standard deviation of three independent biological replicates with each three technical replicates are illustrated.
